# Practicing nurses perspectives of clinical scholarship: a qualitative study

**DOI:** 10.1186/1472-6955-12-21

**Published:** 2013-09-25

**Authors:** Lesley Wilkes, Judy Mannix, Debra Jackson

**Affiliations:** 1 Family and Community Health Research Group (FaCH), School of Nursing and Midwifery, University of Western Sydney and Conjoint Appointment with Nepean Blue Mountains Local Health District, Clinical Nursing Research Unit, Nepean Hospital, PO Box 63, 2751, Penrith, NSW, Australia; 2 School of Nursing and Midwifery, University of Western Sydney, Locked Bag 1797, 2751, Penrith, NSW, Australia; 3 Faculty of Health, Associate Head, World Health Organization Collaborating Centre for Nursing, Midwifery & Health Development, University of Technology Sydney, PO Box 123, 2007, Broadway, NSW, Australia

**Keywords:** Clinical scholarship, Nursing, Research, Practicing nurses

## Abstract

**Background:**

There is a scarcity of research published on clinical scholarship. Much of the conceptualisation has been conducted in the academy. Nurse academics espouse that the practice of nursing must be built within a framework of clinical scholarship. A key concept of clinical scholarship emerging from discussions in the literature is that it is an essential component of enabling evidence–based nursing and the development of best practice standards to provide for the needs of patients/clients. However, there is no comprehensive definition of clinical scholarship from the practicing nurses. The aim of this study was to contribute to this definitional discussion on the nature of clinical scholarship in nursing.

**Methods:**

Naturalistic inquiry informed the method. Using an interpretative approach 18 practicing nurses from Australia, Canada and England were interviewed using a semi-structured format. The audio-taped interviews were transcribed and the text coded for emerging themes. The themes were sorted into categories and the components of clinical scholarship described by the participants compared to the scholarship framework of Boyer [JHEOE 7:5-18, 2010].

**Results:**

Clinical scholarship is difficult to conceptualise. Two of the essential elements of clinical scholarship are vision and passion. The other components of clinical scholarship were building and disseminating nursing knowledge, sharing knowledge, linking academic research to practice and doing practice-based research.

**Conclusion:**

Academic scholarship dominated the discourse in nursing. However, in order for nursing to develop and to impact on health care, clinical scholarship needs to be explored and theorised. Nurse educators, hospital-based researchers and health organisations need to work together with academics to achieve this goal.

Frameworks of scholarship conceptualised by nurse academics are reflected in the findings of this study with their emphasis on reading and doing research and translating it into nursing practice. This needs to be done in a nonthreatening environment.

## Background

Scholarship is elusive. In the academy, scholarship is traditionally measured in terms of original research that is followed by publication in highly ranked and peer-reviewed journals in specialised disciplines. Wise, Retzleff and Reilly [1] suggest scholarship is seen as creative intellectual work that adds to our intellectual history through its communication, and is valued by those for whom it was intended. This is affirmed by Manley, McCormack & Wilson [2] who conceptualised scholarship as work that must be: made public, available for peer review and critique to accepted standards, and be able to be reproduced and built upon by other scholars.

The term 'clinical scholarship’ is one that has been used in nursing discourses over the recent past. Nurse academics have espoused that nursing must build a culture of clinical scholarship [3-5]. Assuming an understanding of this form of scholarship without defining it, O’Neil et al., [6] postulate that 'the cornerstone of clinical scholarship is the transfer of research to practice’ (p73). In this paper we sought to elucidate understandings and develop a contextual definition of clinical scholarship for clinical nursing.

### Scholarship

Discussion on traditional scholarship and its significance in bringing nursing to its essential place in the academy has been apparent in the literature since at least the 1970s [4,5,7,8]. In terms of this literature conceptualisation of scholarship includes: a breadth and depth of discipline knowledge, mastering systematic knowledge, excellence in the discipline, rigorous science, and high levels of comprehension. Cameron-Traub [9] considered scholarship in nursing to include three activities: conceptual processes (rationality; thinking), clinical processes (through practice) and empirical processes (through sensory perception). Scholarship in nursing has also been considered in the light of the seminal work of Boyer [10] when he reconsidered scholarship in the context of the practice disciplines, in particular, in schools and universities. In this framework Boyer proposed four dimensions to scholarship which go beyond research and publication, and can be seen to work separately or be intertwined:

1. Scholarship of discovery investigative work - research.

2. Scholarship of integration giving fuller meaning to isolated facts fitting one’s own research to others into larger intellectual patterns - making connections across disciplines.

3. Scholarship of teaching, creating teaching in a planned and evaluated form.

4. Scholarship of application: how the knowledge can be helpful to society.

Academic exploration has continued on this framework in the teaching discipline and Glassick [11,12] developed six standards with examples for the four forms of scholarship: clear goals, adequate preparation, appropriate methods, significant results, effective communication, and reflective critique. The framework has been taken up by nurses in developing nurse education programs [13], nursing faculty development [14] and engaging with a community [15].

Three frameworks utilising Boyer’s [10] framework as a building block were developed by nurses to reflect scholarship and have been articulated in the literature [15-17]. The essential components of these three frameworks are depicted in Table 1. Jacelon et al’s [15] aim was to conceptualise Boyer’s framework in away more easily comprehended by nurses working in a community program. Storch [16] further explicated Boyer’s framework of scholarship for nursing while working with the nursing faculty at 10 tertiary institutions in Canada. They simplified the four components, giving various examples from nursing. With an emphasis on a discussion of the scholarship of discovery, Thoun [17] developed a theoretical paper arguing that Boyer’s framework was developed to emphasise empirical, positive research with an emphasis on organisational rather than individual goals. This framework moves away from the four strands of discovery, integration, teaching and application to a three element model as explicated in Table 1.

**Table 1:** **Summary of components of nursing scholarship in frameworks described in the literature and closely aligned to Boyer’s**[10]**framework of scholarship for practicing disciplines such as teaching**

**Jacelon *et al*. [**[15]**]**	**Storch & Gamroth [**[16]**]**	**Thoun [**[17]**]**
*● Scholarship of discovery*: knowledge for its own sake creating a venue for research, exploring what is the aesthetic leadership in nursing.	*● Scholarship of discovery*: what is to be known.	*● Emergent scholarship*: innovative research and knowledge where there is a focus on the use of nursing theories and models in research.
*● Scholarship of integration*: connecting academic knowledge base of scholar with clinical knowledge of practicing nurse with the academic knowledge of a scholar.	*● Scholarship of integration:* finding meaning in results and making connections across disciplines.	*● Educational and administrative scholarship:* systematic inquiry into established teaching or administrative process- curriculum development, policy development, evaluation teaching research; e.g. use of clinical simulation.
*● Scholarship of teaching*: creating learning space in clinical area, engage clinical staff and students in learning process.	*● Scholarship of teaching*: should be planned, continuously examined, with clear links between teaching and learning (new knowledge through interaction).	*● Professional scholarship:* inquiry into practice, imaginative artistic and resourceful translating, transforming and pushing borders of practice, the interplay between knowledge and practice policy development, and evaluation of practice leadership.
*● Scholarship of application*: engaging in faculty practice where theory, practice and research inform one another, expert consultation of nursing care.	*● Scholarship of application*: consideration of how knowledge can or could be helpful to society – using concepts theories and principles in nursing practice.	

### Clinical scholarship

There is a scarcity of articles on clinical scholarship. From a series of workshops of with experts and using personal exemplars from practice in the late 1990s Sigma Theta Tau International (STTI) defined clinical scholarship as an approach which enables evidence-based nursing and the development of best practice to meet the needs of clients efficiently and effectively’ [18]. In an attempt to arrive at more definitional clarity, STTI personified it by describing clinical scholars as those nurses who are curious, critical thinkers, reflectors on practice, who develop an environment of sharing the results of their research with the broader nursing and general community [18].

Masson [19] suggests that nurse clinicians may reflect on practice and look up research but not do it. However, Roberts [20] suggests that this maybe clinical scholarship not research scholarship. None-the-less, most authors see clinical scholarship as having components of research. Palmer [21] defines clinical scholarship as the integration of theoretical and experiential knowledge, that is, it encompasses the knowledge and learning derived from the analytical observation of clients and patients (p318). She emphasises that it must include intellectual activity of thinking, analysis and synthesis. Diers [22] extends this definition to include writing – the vital dissemination phase of generating knowledge. These precepts are reinforced again by Schlotfeldt [23] who states that it is 'nursing’s clinical scholarship that must be depended on to generate promising theories for testing that will advance nursing knowledge and ensure nursing’s continued essential service to humankind’ (p8).

In this paper we seek to contribute to the definitional discussion on the nature and characteristics of clinical scholarship in nursing. This paper was drawn from a study that sought to seek an understanding of clinical scholarship from the perceptions of nurse clinicians. The other aspects of clinical scholarship pursued in the study was its enactment by clinical scholars in practice and the similarities and differences of this role to the clinical leader and clinical scholar in nursing and is reported elsewhere [24].

## Methods

This study was grounded in the philosophy of naturalistic inquiry where the central premise is that each individual sees the world differently but from shared and contrasting ideas, meaning of phenomenon can be developed [25]. Using the interactive approach of interviewing the researcher and the participant, co-construct the meaning of a definition of clinical scholarship [3,25]. The study was conducted in three countries – Canada, the United Kingdom (UK) and Australia.

### Participants

Nurses employed in a full-time or part-time clinical capacity and either current students or graduates of post graduate nursing programs were recruited on a voluntary basis for the study. They were accessed drawing on the team’s professional contacts at five universities (one in Australia, one in Canada and three in the UK). The contact at each university sent emails with attached information sheet and consent form to the post graduate students in their faculty and asked them to return a signed consent if they wished to volunteer for an interview. The first author conducted all interviews at a convenient time for the participants at their home university or health facility.

### Data collection

Using a predetermined set of questions relating to what the participants perceived as the components of clinical scholarship, semi-structured interviews were conducted with each participant. Latitude was exercised when necessary by adding more questions to gain more in-depth information and for clarification of facts provided by the participants. All interviews were audio-taped and transcribed as text.

### Data analysis

The text from the interviews was entered into the software program, NVivo [26], to facilitate coding, managing and sorting of data. The coded data were sorted into major categories which reflected the components of clinical scholarship as perceived by the nurse participants. From this interpretation and using exemplars from the analysed text each component is described in the findings. Finally, the components described by the participants were sorted into the component parts of scholarship formulated by Boyer [10].

### Ethical considerations

The study was evaluated and approved by the UWS Human Research Ethics Committee. No identifying features were used in the published text with pseudonyms attributed to the words and opinions of individual participants.

## Results

### Participants

Eighteen clinical nurses participated in this study and a summary of their characteristics are available in detail in Mannix et al., [24]. They worked across three continents from Canada (1, 5.5%), Australia (5, 27.7%), and England (12, 66.6%). Their average age was 42.2 years with a range of 29–67 years while their clinical careers spanned 1.5 to 30 years, with an average of 17 years. The majority worked in acute adult and child care (13, 72.2%). The others worked in palliative care, health department management and pain management. Most completed a certificate or diploma as their first qualification. The most common higher degree was Master of Nursing (10, 55.5%) with four (22.2%) having a PhD.

### Components of clinical scholarship

As discussed in the literature, scholarship is elusive and often difficult to conceptualise and verbalise, and this was apparent in the participants’ preliminary comments on clinical scholarship. For some of the nurse participants in the study, at first sight they viewed scholarship in a simplistic light as financial support, *we get to study* (L3). Others stated: *It’s a difficult question; maybe it’s something nurses presently don’t do… it’s something attached to study at a university* (M3). *Sometimes I think it’s easier to see in an academic environment sometimes it’s more confusing. It’s not clear cut* (C1). This belief that scholarship is only university-based is confirmed again by another participant’s comment: *I didn’t think about scholarship at all when in clinical area but since starting my PhD I think about it quite a bit* (A3). However, the participants generally thought clinical scholarship was important with one participant asserting that *we need it, nursing is still emerging… we need to add more knowledge to be evidence-based practice* (S1). Perhaps, as one participant commented, *we [nurses] haven’t talked about it [clinical scholarship] they talk about it more in school teaching and stuff* (S2).

**Figure 1:**
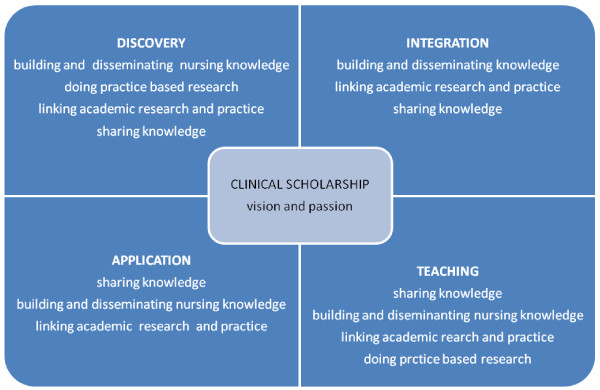
**Major components of clinical scholarship extracted from participants interviews and categorised according to Boyer’s [**[10]**] framework of scholarship.**

The main components of clinical scholarship described by the participants were: building and disseminating nursing knowledge, doing practice-based research, sharing knowledge, and linking academic research and practice. These components are described separately in the section below and are compared to Boyer’s [10] conceptualisation of scholarship in Figure 1.

Vision and passion were seen to be essential elements of all the components of clinical scholarship. *It’s about an intuitive need, its foresight, helping others to realise aims and objectives* (M1)*.* In order to enact this scholarship clinical scholars need to have…

*Vision, because if you don’t, if you’re only talking about what you know there’s no new learning. You have to go to a place where you don’t know and I think that is a sign of a scholar, like if a scholar doesn’t have curiosity what’s the point* (C1).

Clinical scholarship has an essential element of passion which will not only help them to challenge authority but motivate and encourage others to pursue scholarly activities. As one participant related: *She [the scholar] motivated them to read more to do some research, probed them* (S2). In this sense, vision and motivation became inspiration and passion:

*you look something up to impress her [the scholar - a nurse specialist in intensive care] there was about four or five of us who actually formed our own study much to the delight of the Nurse Unit Manager and the Educator* (A1).

Through this vision and passion a clinical scholar *must be able to challenge, shift and rewrite clinical practice and not only do it in practice but in the academic world* (L1). Therefore, as argued by the participants, clinical scholarship is easier for a nurse who has both a clinical, academic/research role. Clinical scholarship is exemplified by one participant’s description of a clinical scholar: *a clinical nurse who is able to bring together many different knowledge bases which would include, research, academia, evidence and practice disciplines in practice* (L1).

### Building and disseminating nursing knowledge

The keystone component of clinical scholarship for all the participants was that knowledge gained by any scholarly endeavour must be made public: *disseminate what you know to abroad audience* (M3). Another participant confirmed this in her definition: *It’s thinking about what you’re doing, it’s clinical audit, doing research, writing it up, disseminating that through meetings and stuff like that, to me that is clinical scholarship* (L1). Like most of the participants, one of them emphasised publications: *I think it’s an obligation to publish is the gold standard if we are thinking about scholarship* (M2). However, the knowledge can be academic scholarship, such as theory and model building, but must be *grounded in practice* (M4) if it is clinical scholarship.

The scholarly activity needs to be disseminated to build and expand nursing knowledge so it is available and used by others: *you see them presenting at conferences, speaking about the papers … people are using them in other intensive care units* (A4). As another participant described, scholarship is*a continuum from clinical practice to publication… been a driver for change in nursing practice* (A3).

### Sharing knowledge

To a lesser degree, the participants emphasised sharing knowledge which was often more informal than public dissemination of knowledge, often a one-to-one communication within an area of practice. The participants felt the scholar was someone who they could go to provide expert consultation on nursing care gained from research.

*You could ask her anything [the scholar – a nurse specialist] if you wanted to know something, she’d be the one to go to and ask. She would definitely know … another nurse I worked with would ask you what you know and then share her’s. So that she tells you what she knows* (A1).

However, some specialists may not be scholars, as they do not share: *they get to a level, they seem to own the knowledge and they could pass on some very pertinent knowledge, you know* (A3).

Clinical teaching was thus the focus of clinical scholarship, but not necessarily as formal teaching programs. Only one participant mentioned a scholar as doing extensive teaching in the clinical area.

### Linking academic research and practice

Linking academic research and practice was a much more formal aspect of clinical scholarship where the scholarly research was translated or transformed into evidence for practice. In some cases the evidence was used to formulate policy and standards of practice: *it’s doing a systematic review of the literature, not just choosing what you like, but actually looking at it and trying to incorporate it into your policy various grades of evidence* (L2).

Clinical scholarship must be valued by those for whom it was intended. As one participant commented, i*t* was *promoting clinicians at the bedside to change practice, you know, based on what was published, what was shown to be better… being involved in the change* (A1). For the participants clinical scholarship occurs when: *the knowledge from research is transferred directly into practice… for example, what safety issues existed and these were incorporated into mandatory training which we do* (L1).

Nurses can also learn and begin their journey to their own clinical scholarship through their involvement in clinical medical trials*:*

*They understand research through their basic degree; they learn about research through the trials, about recruitment, randomisation and getting consent which is really important and really difficult up here in ICU* [Intensive Care Unit]*… although not primarily responsible for design and analysis they see the protocol… they can think they could do something. They could do a study on you know* (L2).

### Doing practice-based research

In order to achieve a link between academic research and practice, clinical scholarship must be directed at practice-based research. It is the discovery and building of nursing knowledge through creative, experiential and experimental work in practice. Clinical nurses need to be exposed and encouraged to do their own or collaborative research to build the knowledge base of nursing. However, they require the necessary skills, but sometimes:

*In nursing we tend to segregate research to academics, teaching to educators, practice specialities to clinicians; they are motivated to nurse not research, however, they can develop their expertise in collaborative research with the academic … we cannot just be in one sphere we need to share and do research and share our clinical knowledge to others* (M2).

Clinical scholarship involves identifying issues in practice and beginning *to explore how we can address the uncertainties that we face in practice today* (L1). As one participant described:

*She* [clinical scholar] *developed tools, she is researching the practice she has developed, and you spend a lot of years doing this. For a long time she did this with academic qualifications, she has a PhD now but certainly I would visualise this as someone clinically engaged in clinical scholarship* (M2).

## Discussion

This study is unique in that it is the first time clinicians have provided their understandings of clinical scholarship in nursing. Much of the previous research and conceptualising has been conducted within the academy. Vision and passion to motivate and encourage others to develop were seen by the clinicians as essential to all components of clinical scholarship. It is the adhering cement of clinical scholarship. This vision and passion extends into pushing boundaries, providing leadership in clinically focussed research and linking research to practice. This has been highlighted as a key to scholarship [27-29] and indeed clinical scholarship [13,21,30,31].

As there is no framework to describe clinical scholarship published in the literature the components of clinical scholarship described by the practicing nurses in this study were compared to Boyer’s generic model of scholarship. All components espoused by the nurses can be seen to reflect the four dimensions of scholarship described by Boyer [10]. However, the vision and passion highlighted in the present study as an essential underpinning linchpin of the framework is not at the cornerstone of Boyer’s organisational needs-focused framework.

If the three frameworks for nursing scholarship (Table 1) are examined, it can be seen that both the Jacelon et al. [15] and Storch and Gamroth, [16] models, which follow Boyer’s [10] construct, are reflected in the findings of this current study. If the framework of Thoun [17] is compared to the current findings, it is apparent that while emergent and educational/administrative scholarship are evident, the emphasis on the clinicians’ understanding of clinical scholarship is most closely linked to professional scholarship. This emphasis may describe the uniqueness of this scholarship within the nursing context. As revealed in the current study, clinical scholarship develops from learning from other’s research, reading research and putting research findings into practice, systematic reviews, developing the scholar nurse’s own research, developing collaborative research and doing research from the scholar’s own practice base. This reflects other literature on the development of clinical scholarship [18,23,31].

In order to develop and nurture clinical scholarship, an enabling research culture within health and the academy must be developed to capture the creativity of the clinician. This culture needs to be characterised by research productivity, positive collegial relationships, inclusivity and non-competitiveness, and effective research processes and training. The health arena must provide a safe environment for clinicians to discuss and theorise about clinical scholarship [18,32]. As well, health organisations and nurse leaders in these organisations must encourage and build structures such as practice development projects with a focus on improving patient care [2,4,28,33] by encouraging and assisting clinical nurses to pursue research and its translation back to practice.

### Limitation of the study

This qualitative study is limited by the number of participants. However, the participants, in sharing their world view of clinical scholarship will assist the profession of nursing to think about a construct which is often seen as unimportant in the clinical arena and is shuffled to the academy. As Lincoln and Guba [25] have challenged the research world in their pursuit of naturalistic inquiry in research (constructivism), each of us sees the world differently but our combined perceptions, even that of a few, often share similarities and differences which enhance our understanding of phenomena. This study has been worthwhile in exploring and extending our understanding of clinical scholarship with the busy contemporary world of nursing practice.

## Conclusion

The concept of clinical scholarship should be a cornerstone of nursing as a profession and as a discipline. However, currently, scholarship in the academic realm dominates the literature. In this study we have shown the importance of theorising clinical scholarship for the future development of nursing and the pursuit of continued improvements to health care. We believe there is a clear role for educators and hospital-based researchers to assist in generating understandings about clinical scholarship and what it means in practice. There is a need to create spaces for nurses to consider the nature of clinical scholarship, and how it could be enacted in the clinical realm. In this way a further and vital contribution to the future of the discipline can be made, and a real commitment to excellence in clinical practice can be demonstrated.

## Competing interests

The authors declare that they have no competing interests.

## Authors’ contributions

All authors read and approved the final manuscript.

## Pre-publication history

The pre-publication history for this paper can be accessed here:

http://www.biomedcentral.com/1472-6955/12/21/prepub
